# The RS4939827 polymorphism in the *SMAD7* GENE and its association with Mediterranean diet in colorectal carcinogenesis

**DOI:** 10.1186/s12881-017-0485-5

**Published:** 2017-10-30

**Authors:** Jéssica Alonso-Molero, Carmen González-Donquiles, Camilo Palazuelos, Tania Fernández-Villa, Elena Ramos, Marina Pollán, Nuria Aragonés, Javier Llorca, M. Henar Alonso, Adonina Tardón, Pilar Amiano, José Juan Jiménez Moleon, Rosana Peiró Pérez, Rocío Capelo, Antonio J. Molina, Inés Gómez Acebo, Marcela Guevara, Beatriz Pérez Gómez, Virginia Lope, José María Huerta, Gemma Castaño-Vinyals, Manolis Kogevinas, Victor Moreno, Vicente Martín

**Affiliations:** 1 Grupo de Investigación en Interacciones Gen-Ambiente y Salud. Instituto de Biomedicina (IBIOMED), Universidad de León, León, Spain; 2 Centro de Investigación Biomédica en Red (CIBERESP) and Oviedo University; Departamento de Ciencias Biomédicas. Universidad de León. Campus de Vegazana, León, Spain; 3 Universidad de Cantabria, Santander, Spain; 4 CIBER Epidemiología y Salud Pública (CIBERESP), Madrid, Spain; 5 Cancer and Environmental Epidemiology Unit, National Centre for Epidemiology, Carlos III Institute of Health, Madrid, Spain; 6 Cancer Prevention and Control Program, Catalan Institute of Oncology, Hospitalet de Llobregat, Barcelona, Spain; 7 Department of Clinical Sciences, Faculty of Medicine, University of Barcelona, Barcelona, Spain; 8 Colorectal Cancer Group, Bellvitge Biomedical Research Institute (IDIBELL). Hospitalet de Llobregat, Barcelona, Spain; 9 Oncology Institute IUOPA, Universidad de Oviedo, Oviedo, Asturias Spain; 10 Instituto de Salud Pública de Navarra, Pamplona, Navarra Spain; 11 Instituto de Investigación Biosanitaria de Granada (ibs.GRANADA), Hospitales Universitarios de Granada/Universidad de Granada, Granada, Spain; 12 Dirección General de Salud Pública, Fundación para el fomento de la investigación sanitaria y biomédica de la Comunidad Valenciana, FISABIO-Salud Pública, Barcelona, Spain; 13 Centro de Investigación en Salud y Medio Ambiente (CYSMA), Universidad de Huelva, Huelva, Spain; 14 Cancer Epidemiology Research Group, Oncology and Hematology Area, IIS Puerta De Hierro, Madrid, Spain; 15 Department of Epidemiology, Murcia Regional Health Council, IMIB-Arrixaca, Murcia, Spain; 16 ISGlobal, Centre for Research in Environmental Epidemiology (CREAL), Barcelona, Spain; 17 IMIM (Hospital del Mar Medical Research Institute), Barcelona, Spain; 18 Universitat Pompeu Fabra (UPF), Barcelona, Spain; 19 School of Public Health, Athens, Greece; 20 Grupo de Investigación en Interacciones Gen-Ambiente y Salud de la Universidad de León, León, Spain

**Keywords:** Colorectal cancer, *SMAD7*, rs4939827, Mediterranean diet, Gene-environment

## Abstract

**Background:**

The objective of our investigation is to study the relationship between the rs4939827 SNP in the *SMAD7* gene, Mediterranean diet pattern and the risk of colorectal cancer.

**Methods:**

We examined 1087 cases of colorectal cancer and 2409 population controls with available DNA samples from the MCC-Spain study, 2008–2012. Descriptive statistical analyses, and multivariate logistic mixed models were performed. The potential synergistic effect of rs4939827 and the Mediterranean diet pattern was evaluated with logistic regression in different strata of of adherence to the Mediterranean diet and the genotype.

**Results:**

High adherence to Mediterrenean diet was statistically significantly associated with colorectal cancer risk. A decreased risk for CRC cancer was observed for the CC compared to the TT genotype (OR = 0.65 and 95% CI = 0.51–0.81) of the rs4939827 SNP Also, we could show an association between the Mediterranean diet pattern (protective factor) and rs4939827. Although the decreased risk for the CC genotype was slightly more pronounced in subjects with high adherence to Mediterrenean diet, there was no statistically significant synergistic effect between genotype CC and adherence to the Mediterranean dietary pattern factors.

**Conclusion:**

The SMAD7 gene and specifically the allele C could be protective for colorectal cancer. An independent protective association was also observed between high adherence Mediterranean diet pattern and CRC risk. Findings form this study indicate that high adherence to Mediterranean diet pattern has a protective role for CRC cancer probably involving the Tumor Growth Factor- β pathway in this cancer.

**Electronic supplementary material:**

The online version of this article (10.1186/s12881-017-0485-5) contains supplementary material, which is available to authorized users.

## Background

Colorectal cancer (CRC) has the third highest incidence in men, and the second highest in women worldwide [1]. This cancer has an increasing incidence in developing countries [2] probably due to the increasing prevalence of environmental factors that contribute to the development of CRC, for instance, dietary pattern and physical activity [3]. On the other hand, mortality levels are decreasing in countries with specialized care and better screening services [4]. Gene-Environment (GxE) interactions may play an essential role in increasing the susceptibility to developing colorectal cancer [3]. Among these factors, it is important to pay attention to the Mediterranean Diet Pattern adherence, since lower adherence to this pattern is more common as a direct consequence of the general westernization of the lifestyle’s population [5–7]. Assessing the adherence to a Mediterranean dietary pattern is one of the particularly interesting approaches which may help to understand the relationship between diet and CRC [8]. However, the effect of this pattern over health depends on many individual aspects, involving genetic factors and polymorphisms. The lack of knowledge about the interactions between diet-polymorphism is a huge problem for the public health [9].

**Fig. 1:**
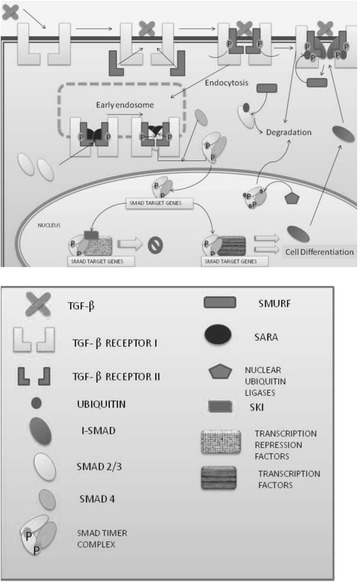
Signaling pathway TGFβ-SMAD

Earlier studies show a possible relationship between the diet pattern and the rs4939827 SNP in *SMAD7* (*SMAD family member 7*), which has been associated to CRC previously. The *SMAD7* gene acts as a Transforming Growth Factor Beta (TGF-β) family inhibitor by blocking the pathway signaling (Fig. 1) [10]. *TGF-β* encodes for a cytokines family, which are multifunctional peptides that control some process like cell proliferation. In this way, when TGF-β is inhibited by the interaction with SMAD7, cell proliferation is promoted and this could lead to the development of cancer. Another key gene in the undirected regulation of *SMAD7* expression is the gene *Ski (SKI proto-oncogene)*, which acts by blocking the *TGF-β* target genes, as *SMAD7* [11, 12].

The interaction between the Mediterranean diet and the *SMAD7* gene may be due to the action of other genes that are involved in the TGF-β pathway, in particular *SMAD3 (SMAD family member 3)*, which is inhibited by the first one [13]. This pattern of diet is characterized by high fiber contents and before a fermentative process Na-butyrate is produced. This compound interacts with *SAMD3*, highlighting the signaling of this pathway [11]. In this way, we can check that the relationship between the dietary pattern and *SMAD7* expression is real.

The goal of this article is to investigate the relationship between the rs4939827 SNP, the Mediterranean Diet Pattern and the risk of CRC.

## Method

### Study population

MCC-Spain is a multicentric case-control study with population controls aiming to evaluate the influence of environmental exposures and their interaction with genetic factors in common tumors in Spain (prostate, breast, colorectal, gastroesophageal and chronic lymphocytic leukemia). Between September 2008 and December 2013, subjects between 20 and 85 years of age (matched by 5-year age interval) with histologically-confirmed newly-diagnosed colon or rectum cancer (ICD-10: C18, C19, C20, D01.0, D01.1, D01.2) were recruited in 23 Spanish hospitals from 12 Spanish provinces. Simultaneously, population-based controls frequency-matched to cases, by age, sex and region with the join distribution of the tumors included in MCC were randomly selected from primary care centers within hospitals’ catchment areas. All participants signed an informed consent. Approval for the study was obtained from the ethical review boards of all recruiting centers. Additional information regarding the study design is provided elsewhere [14].

In our study, 3496 individuals were included from this MCC-Spain study. For this analysis, 1087 cases of colorectal cancer and 2409 controls were involved with available DNA samples for genetic analysis.

### Lifestyle information

A computerized epidemiological questionnaire including self-reported socio-demographic and anthropometric data, family history of cancer, environmental exposures, use of selected drugs, reproductive history and current and past lifestyle behaviors (including leisure time physical activity and sedentary lifestyle) was administered by trained personnel in a face-to-face interview at enrolment. Waist and hip circumferences were measured by the interviewer [14]. Subjects were provided a previously validated semi-quantitative Spanish Food Frequency Questionnaire (FFQ) [15] which was modified to include regional products. The FFQ included 140 food items, and assessed usual dietary intake during the previous year. The FFQ included the specific cooking methods for meat and some pictures to establish how thoroughly-cooked participants prefer it. The FFQ was self-administered and returned by mail or filled out face to face (global response rate 88%). Frequency data was used to derive amount (g/day, g/1000 kcal) of each of the individual food types and summary variables. The food composition table has been a compiled table from the Centre for Higher Studies in Nutrition and Dietetics (CESNID) and other specific sources [16]. Cross-check questions on food groups intakes were used to adjust the frequency of foods eaten and reduce misreporting of food groups with large numbers of items [17, 18]. Data from this questionnaire was used to obtain a score in order to know the adherence to the Mediterranean diet by Sofi F [19]. Sofi F. et al. have computed the adherence to this type of diet taking into account 9 groups of foods (vegetables, legumes, meat, fish, integral cereals, fruit, dairy products, alcohol, olive oil) [19]. The range of this score goes from zero to eighteen points. From that score, we create subsets based on individuals with low (if the score is <9), medium (from 9 to 11 points) or high (if the score is higher than eleven points) adherence to this kind of diet [12]. Since the Mediterranean diet has a high fiber content and the *SMAD7* gene can interact with it, an analysis of this factor was considered essential.

For the variable of physical activity, the last 10 years were taken into account. This parameter also includes recreational physical activity. The assessed parameter was METs, a measured unit of metabolism. The responses obtained allowed us to create four subpopulations: sedentary, low physical activity, average physical activity and intense physical activity.

### Samples processing

Peripheral blood (27 ml) was drawn from participants, which were aliquoted in whole blood, plasma, cellular fraction for DNA extraction, and serum and stored at −80 °C. Saliva was collected for subjects refusing to donate blood with the Oragene® DNA Kit and stored at room temperature until DNA extraction. We collected biological samples for DNA extraction for participants with interview, as well as toenail and hair samples. In 4 centers (Madrid, Cantabria, Asturias and Huelva) cases and controls also donated urine samples (60 ml) that were aliquoted and frozen at −80 °C. Fresh tumor biopsies or paraffin embedded samples are available in all participating hospitals. Standardized basic clinical and pathological information on the diagnosis and treatment of tumors was collected from hospital records by using a predefined format.

Genetic analyses were carried out within MCC-Spain and also through participation in international consortia. The InfiniumHumanExomeBeadChip from Illumina was used to genotype >200,000 coding markers plus 6000 additional custom variants on the pathways of interest [14].

### SNP selection

After a literature search, different SNPs that were associated with CCR were selected. These SNPs were processed with the PLINK software in order to verify that they had been collected in the database of the MCC-Spain. This file contains the basic data needed for statistical analysis (cases and controls, SNPs, and the identifier of each individual). Finally, after an ad-hoc evaluation of the polymorphisms, rs4939827 was selected and we performed the statistical analyses described in the following section because only this SNP was considered relevant in its relation with colorectal cancer (see Additional file 1: Table S1).

### Statistical methods.

First, a descriptive statistical analysis was performed to determine the characteristics of our study population. For age, mean and standard deviation were calculated. For the rest of variables, we calculate frequencies of cases and controls using STATA. Table 1 show variables used in analysis and how they were classified.

**Table 1:** Descriptive analysis results. Characteristics of cases and controls

		CONTROLS		CASES		*p*-values
		N	%	N	%	
		2409		1087		–
Age	(Mean/SD)	63.13	11.5	66.64	10.4	–
Sex	Men	1300	54	708	65.1	<0.0001
	Women	1109	46	379	34.9	
First Degree Family History	No	2081	90.6	855	82.1	<0.0001
	Yes	216	9.4	186	17.9	
Cultural level	Less than primary school	409	17	288	26.5	<0.0001
	Primary school	803	33.3	458	42.1	
	Secundary school	698	29	221	20.3	
	University	499	20.7	120	11	
BMI	Normal weight	759	31.5	259	23.8	<0.0001
	Less than normal weight	23	1	13	1.2	
	Overweight	873	36.2	399	36.7	
	Obesity	754	31.3	416	38.3	
Total energy (Kcal/Day)	Low	795	33	296	27.2	<0.0001
	Medium	794	33	320	29.4	
	High	820	34	471	43.3	
METS	Sedentarism	855	35.5	468	43.1	<0.0001
	Low physical activity	385	16	134	12.3	
	Moderate physical activity	313	13	109	10	
	High physical activity	856	35.5	376	34.6	

*N* Number, *BMI* Body Mass Index, *METS* Metabolic Equivalent of Task. The cut point for category of total energy are <1586.3 Kcal/day, ≥ 1586.3 Kcal/day & < 2046.4 Kcal/day and ≤2046.4 for low, medium and high level respectively.For the physical activity, the cut points are 0 when the there is no physical activity, < 8 METS*hours/week for low physical activity, ≥ 8 and < 16 METS*hours/week for moderated physical activity and >16 METS*hours/week for high physical activity (according to American College of Sport Medicine)

Then multivariate logistic mixed models, including the study region as a random effect term, were performed to evaluate the association between genotypes of selected polymorphisms and the risk of CRC. The same analysis was made in order to show the association of the component of the Mediterranean diet with the colorectal cancer. Adjusted odds ratios and confidence intervals at 95% were calculated by reference to the homozygous genotype of the most common allele (T allele). The minimally adjusted odds ratio was calculated by adjusting by sex, age and educational background. The adjusted odds ratios were calculated taking into account the variables mentioned before and BMI, total energy intake, family history first degree, race and METS.

Finally, linear regression was used for associations between polymorphisms and the study variables. For this, the online software used was SNPstats, by the log-additive model mainly, although we also observed the results of other models (dominant, codominant, recessive and overdominant) [20]. By using STATA, the rs4939827 SNP association with the Mediterranean diet pattern was evaluated with logistic regression. For that, we stratified the variable of adherence to the Mediterranean diet and the genotype was also taken into account.

## Results

**Fig. 2:**
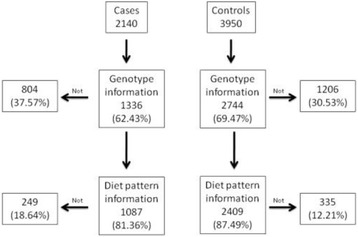
Algorithm for cases and controls for this study, selected from the population which participated in the project

The initial study population consists of 6090 individuals, distributed into 2140 cases and 3950 controls. Among them, 3496 had both genetic information and the dietary pattern available, the algorithm of missing data can be seen in Fig. 2. Table 1 shows the main characteristics of those individuals which the analysis was performed with. Analyzing the missing data, it has been observed that only men have significant differences (in both case and control groups). The rest of variables do not show significant differences (Data not shown).

### Descriptive analysis

In this study 3496 participants have been included, 1087 cases (65.1% males; 34.9% females) and 2407 controls (54% males; 46% females). The characteristics of the individuals of this study are described in Table 1. Data belong to a multi case-control study and matches were performed by frequencies in all types of tumors (colorectal, mama, prostate, leukemia, stomach), so it can be the reason for the mismatch between the sex distribution between affected cases and frequency-matched controls.

In the study population, the average age is 66.6 (±10.4) in cases and 63.1 (±11.5) in controls. Physical activity habits show a sedentary population, with 43.0% in cases and 35.5% in controls who do not practice physical activity. BMI shows that only 23.8% in cases and 31.5% in controls have an adequate weight. Most of the population suffers from obesity or being overweight.

### Mediterranean diet pattern and components analysis

The components of the Mediterranean diet pattern by case-control status are shown in Table 2. We observe that there are significant differences in alcohol intake in the last 10 years between cases and controls, however we have not got any clear evidence that alcohol intake in the Mediterranean diet is associated with CRC. The opposite case can be found in vegetables and legumes, where a high intake of them may be protective against colorectal cancer. On the other hand, meat and dairy products are risk factors when they are consumed in high quantities. Other components (integral cereals, fish and olive oil) do not show significant differences. Fruit cannot be analyzed because there are not enough subjects (Table 2).

**Table 2:** Association between intake levels of food components in the Mediterranean diet anc case (affection) status. The cut points are based on the score criteria for Sofi’s Mediterranean diet pattern

		CONTROLS		CASES		OR minimally adjusted	CI (95%)	OR adjusted	CI (95%)
		N	%	N	%				
		2409		1087					
Alcohol	Moderate intake (12 g/day-24 g/day)	612	25.4	386	35.5	1	1	1	1
	Hight intake (>24 g/day)	1471	61.1	537	49.4	1.17	0.92–1.49	0.76	0.60–0.97
	Low intake (<12 g/day)	326	13.5	164	15.1	0.77	0.61–0.98	1.04	0.81–1.33
Vegetables	Low intake (<100 g/day)	563	23.4	306	28.2	1	1	1	1
	Moderate intake (100 g/day-250 g/day)	1378	57.2	624	57.4	0.82	0.68–0.98	0.85	0.70–1.03
	Hig/dayh intake (>250)	468	19.4	157	14.4	0.54	0.42–0.70	0.55	0.42–0.72
Legumes	Low intake (<70 g/day)	1561	64.8	670	61.6	1	1	1	1
	Moderate intake (70 g/day-140 g/day)	383	15.9	177	16.3	0.94	0.76–1.16	0.97	0.77–1.21
	Hig/dayh intake (>140 g/day)	465	19.3	240	22.1	0.75	0.60–0.92	0.77	0.61–0.96
Meat	Low intake (<80 g/day)	185	7.7	152	14.0	1	1	1	1
	Moderate intake (80 g/day-120 g/day)	442	18.3	236	21.7	1.4	1.15–1.70	1.29	1.05–1.60
	Hig/dayh intake (>120 g/day)	1782	74.0	699	64.3	1.98	1.54–2.55	1.74	1.32–2.30
Dairy products	Low intake (<180 g/day)	1541	64.0	693	63.8	1	1	1	1
	Moderate intake (180 g/day-270 g/day)	557	23.1	250	23.0	0.95	0.74–1.23	0.97	0.74–1.28
	Hig/dayh intake (>270 g/day)	311	12.9	144	13.2	1.02	0.82–1.29	1.01	0.79–1.30
Fruits	Low intake (<150 g/day)	0	0	0	0	1	1	1	1
	Moderate intake (150 g/day-300 g/day)	0	0	0	0	0	0	0	0
	Hig/dayh intake (>300 g/day)	2409	100	1087	100	omitted		omitted	
Cereals	Low intake (<130 g/day)	2327	96.6	1047	96.3	1	1	1	1
	Moderate intake (130 g/day-195 g/day)	71	2.9	32	2.9	0.97	0.62–1.51	1.08	0.68–1.74
	Hig/dayh intake (>195 g/day)	11	0.5	8	0.7	1.57	0.62–4.01	1.42	0.50–4.02
Fish	Low intake (<100 g/day)	169	7.0	91	8.4	1	1	1	1
	Moderate intake (100 g/day-250 g/day)	663	27.5	304	28.0	0.91	0.67–1.22	0.9	0.65–1.25
	Hig/dayh intake (>250 g/day)	1577	65.5	692	63.7	0.82	0.62–1.09	0.84	0.62–1.16
Olive oil	Low intake (<0.1 g/day)	115	10.6	245	10.2	1	1	1	1
	Moderate intake (0.1 g/day-0.99 g/day)	206	19.0	513	21.3	0.88	0.72–1.07	0.85	0.69–1.05
	Hig/dayh intake (>1 g/day)	766	70.5	1651	68.5	0.85	0.67–1.10	0.91	0.70–1.21

The minimally adjusted odds ratio (OR) was calculated adjusting by sex, age (as continuous variable) and educational level from a multivariate logistic mixed model.Area was used as random variable. The adjusted odds ratios were calculating taking into account the variables mentioned before and BMI, total energy intake, family history fist degree, race and METS. (N: Number; CI: Confidence Interval). Cut-offs are based on score criteria of Sofi’s Mediterranean Pattern [19]

Taking into account the adherence to the Mediterranean dietary pattern, 68.6% of cases and in 75.3% of controls have a high or medium adherence to the Mediterranean dietary pattern. All these variables show significant differences between cases and controls (Table 3).

**Table 3:** Association between SNP rs4939827 and the Mediterranean dietary pattern adherence in relation with colorectal cancer taking into account the genotype

	Cases		Controls		Adjusted OR	
	n	%	n	%	OR	CI 95%
Low	341	31.4	594	24.7	1	–
TT	110	32.2	171	28.8	1	–
CT	169	49.6	302	50.8	0.83	0.59–1.17
CC	62	18.2	121	20.4	0.70	0.44–1.08
Medium	431	39.7	1010	41.9	0.85	0.70–1.03
TT	154	35.7	316	31.29	1	–
CT	211	49	478	47.2	0.87	0.65–1.15
CC	66	15.3	216	21.38	0.64	0.44–0.93
High	315	29.0	805	33.4	0.64	0.51–0.79
TT	123	39.1	242	30.1	1	–
CT	144	45.7	405	50.3	0.75	0.55–1.02
CC	48	15.2	158	19.6	0.63	0.42–0.96

CT vs TT: Adjusted Odd Ratio (OR) = 0.82 and 95% Confidence Interval (CI) = 0.69–0.98

CC vs TT: OR = 0.65 and 95% CI = 0.51–0.81

The adjusted odds ratios (OR) was calculating taking into account the variables sex, age, educational level, BMI, total energy intake, family history fist degree, METS. Area was used as random variable (N: Number; CI: Confidence Interval)

### Associations of rs4939827 with CRC and other factors

The rs4939827 SNP was statistically significantly associated with colorectal cancer according to several models. The most important result is referred to the Log-Additive model (*p*-value = 1.00E-04). The association of this polymorphism with CRC can also be observed in other models in a significant way: codominant model (*p*-value = 2e-04); dominant model (*p*-value = 6e-04); recessive model (*p*-value = 8e-04). The analysis of association of this polymorphism with risk factors is also shown in the summary table.

### rs4939827 associations with risk factors of this study

After analyzing the association between rs4939827 and risk factors, statistically significant results can be observed in the case of the degree of adherence to the Mediterranean dietary pattern. Other factors assessed did not show a significant association with the SNP (data not shown). We conducted an analysis for rs4939827 with an interaction term to evaluate the independent main effect of the Mediterranean diet and main effect of the protective rs4939827 allele (see Additional file 2: Table S2). However, none of the results provide a significant *p*-value although the overdominant model is close to *p*-value = 0.05 (*p*-value = 0.07), determining that C allele it the dominant one.

**Fig. 3:**
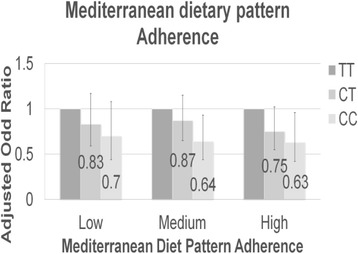
Association between SNP rs4939827 and the Mediterranean dietary pattern adherence in relation with colorectal cancer taking into account the genotype. Reference: TT genotype. Vertical axis: Adjusted Odd Ratio (The adjusted odds ratios (OR) was calculating taking into account the variables sex, age, socioeconomic level, BMI, total energy intake, family history fist degree, METS and area was used as random variable). Abscissa axis: Mediterranean Diet Pattern Adherence

The CC genotype (16.1% in cases and 20.5% in controls) seems to be protective compared to the TT (35.6% in cases and 30.3% in controls) genotype (ORa TTvsCT = 0.82 IC (95%) = 0.69–0.98; ORa TTvsCC = 0.65 IC (95%) = 0.51–0.81). When we check the association with adherence pattern to the Mediterranean diet (only statistically significant association), it can be observed that high adherence is a protective factor (Fig. 3 and Table 3). However, we could not see a synergistic effect between both genotype CC and adherence to the Mediterranean dietary pattern factors.

## Discussion

*SMAD7* is a target gene of the signaling pathway TGFβ-SMAD. For the *TGFβ* expression, it must be joined with its receptor type II. This binding stimulates the receptor type I, which binds to the above complex and, the complete assembly, is able to regulate the formation and activation (by phosphorylation) of the heterodimeric complex SMAD2/3. After the activation, this complex joins SMAD4 (SMAD family member 4) protein, which helps the complex translocation to the nucleus. In this organelle, the activation of the expression of target genes of *TGFβ* occurs, including the *SMAD7* gene and other genes responsible for proliferation inhibition and cell division. This happens when the SKI protein is not in the nucleus, which is capable of binding with the heterotrimeric complex and inhibit its function, inhibiting the expression of the *SMAD7* gene in this way (Fig. 1) [6, 7].

In this study of 1087 cases of CRC and 2409 controls, the rs4939827 SNP with the CC genotype was associated with a reduction of the risk for colorectal cancer. This may be due to *SMAD7* participation modulating the TGFβ pathway. SMAD7 protein is capable of binding with TGFβ, causing its ubiquitination and destruction. In this way, the action of TGFβ is inhibited and cell proliferation can occur. To clarify the role of *SMAD7* in cell growth in the CRC, Halder et al. [21], overexpressed this gene. This fact produces a higher cell growth than in normal conditions (21). At the same time, it has been observed that inhibition of *SMAD7* with a specific oligonucleotide reduces cell growth in CRC [22]. This data leads to the hypothesis that rs4939827 with genotype CC is a protective genotype because the correct expression of the *SMAD7* gene is inhibited, preventing cell proliferation and reducing the susceptibility to colorectal cancer.

This hypothesis is supported by B. Zhang et al. (2014) [23] who share the idea that the gene variant rs4939827 may be associated with an increase of CRC survivorship [23]. Similarly, Slattery M. et al. (2010) [24] also agree with our results as they show that the CC genotype is inversely associated with the risk of colon cancer. Thus, they conclude that individuals containing the homozygous recessive gene variant rs4939827 show a reduced risk of colon cancer by a 27% [24]. Thompson C.L. et al. (2009) [25] performed an analysis of the SNP taking into account the dominant model, stratifying the population according to gender. They noticed that women with the C allele were associated with a decreased risk of CRC. In contrast, they did not obtain significant results in men [25].

The CC genotype of this polymorphism was also associated with survival of other cancers. For example, according to Geng, T.T. et al. (2015) [26], the dominant model showed that the rs4939827 polymorphism was significantly associated with a decreased risk of esophageal cancer by 0.67 fold due to a possible inhibition of the *SMAD7* gene [26].

The T allele must also be taken into account. Although T allele does not show a significant risk in our results, many authors support the idea by Jung KJ et al. (2015) [27], which defended the hypothesis that the T allele of this SNP was a risk factor for colorectal cancer and rectal cancer [27]. Furthermore, according to analysis made by Baert-Desurmont S. et al. (2015) [28] an increased risk is observed depending on the number of T alleles present in genotype [28]. Ho J. W. et al. (2011) [29] analyzed the risk of TC heterozygous and TT homozygous genotypes. They identified both as risk genotypes, but only the homozygous genotype showed a significant result [29]. Tenesa A. et al. (2008) [30], after taking into account the location of the tumor to perform the analysis, noted that the T allele is a risk factor mainly for rectal cancer and not for the colon cancer [30]. In our results, we cannot verify that information, since we did not identify the T allele as a risk allele, only the C allele could be identified as a protective allele. Tenesa A. et al. (2010) [31] also associated the T allele of the rs4939827 SNP with an increase of mortality [31]. Passarelli M.N. et al. (2011) [32] found that *SMAD7* variants that inhibit TGFβ completely may reduce its tumor suppressor activity (resulting in an increased risk), but can also reduce their ability to promote their metastatic promoter activity (resulting in a slower progression of the tumor and improved survival) [32]. Finally, Yao et al. (2015) [33] support that the T allele increases the risk of CRC in Caucasian population [33].

If we analyze the SNP association with diet, our results are statistically significant. However, few authors refer to the association of this factor with the rs4939827 polymorphism. As it is mentioned before, association with the diet is due to the TGFβ signaling pathway genes that interact with *SMAD7*. There is evidence that this association may be due to the ingestion of high amounts of fiber in the Mediterranean diet. Vegetables and fruit, for example, are highly fermentable fiber sources, while products such as wheat bran are low fermentable. The difference is the amount of short-chain fatty acid formed after fermentation of the fiber by the colon’s bacteria in this organ, as described by Nguyen K.A. et al. (2006) [11]. So, food containing fermentable fiber (fruit and vegetables) will produce higher amount of fatty acids than that containing short chain poorly fermentable fiber. Therefore, there is a positive relationship between the consumption of large amounts of fiber and a lower risk of colon cancer. This may be due to the fact that the Na-butyrate (Na-B, the main product of the fermentation of fiber) is able to induce cell cycle arrest, cellular differentiation and, even, apoptosis. It seems that the Na-B is capable of inducing, selectively, Smad3 phosphorylation, so pathway activity of TGF-β is enhanced [6, 7]. Despite this, we have not found a statistically significant interaction in our results. It may be due it has not got enough statistical power or the actual interaction is carried out with SMAD3. This produces that TGF-β is not ever ubiquitined and degraded, so cyclins jogging cell proliferation (p15, p21 and p27) are expressed [34]. Besides, one of *SMAD7* functions is to produce cell cycle arrest in G1 phase. However, because of this mutation, this activity cannot be carried out by this process. Na-B intake helps this action be covered, further minimizing the risk of uncontrolled cell proliferation.

One limitation in this work is that genotype x diet effect could be due to fiber intake rather than adherence to the Mediterranean diet, although both concepts are related.

## Conclusions

There is an important role of SMAD7 gene in colorectal cancer as the allele C is protective against this disease. Also, we can see a protective association between rs4939827 SNP and high adherence to the Mediterranean diet pattern and we suppose that high adherence to Mediterranean diet pattern probably has a protective role. So they may participate together in the TGFβ pathway in colorectal cancer.

## Additional files

Additional file 1: Table S1. Analysis of SNPs related to CRC. The minimally adjusted odds ratio (OR) was computed adjusting by sex, age, educational level. Area, places where cases and controls were recruited, was used as random variable. Association is evaluated for carrying 1 or 2 SNP minor frequency alleles relative to a reference of zero SNP minor frequency allele. (DOCX 22 kb)

Additional file 2: Table S2. Associations between polymorphisms and the Mediterranean Diet Pattern of Sofi considering different genetic models. Genetic models: codominant, dominant, recessive, overdominant, log-additive. (DOCX 16 kb)

## Abbreviations

CESNID: Centre for Higher Studies in Nutrition and Dietetics
CRC: Colorectal cancer
FFQ: Spanish Food Frequency Questionnaire
GxE: Gene-Environment
Na-B: Na-butyrate
ORa: Odd Ratio adjusted
*Ski*: *SKI proto-oncogene*
*SMAD3*: *SMAD family member 3*
SMAD4: SMAD family member 4
*SMAD7*: *SMAD family member 7*
SNP: Single Nucleotide Polymorphism
TGF-β: Transforming Growth Factor Beta
