# Supplementary material for: The RS4939827 polymorphism in the SMAD7 GENE and its association with Mediterranean diet in colorectal carcinogenesis
Source: BMC Med Genet. 2017 Oct 30;18:122. doi: 10.1186/s12881-017-0485-5 (PMC5661920; doi:10.1186/s12881-017-0485-5)
Supplement: Supplementary file 1 — Analysis of SNPs related to CRC. The minimally adjusted odds ratio (OR) was computed adjusting by sex, age, educational level. Area, places where cases and controls were recruited, was used as random variable. Association is evaluated for carrying 1 or 2 SNP minor frequency alleles relative to a reference of zero SNP minor frequency allele. (DOCX 22 kb) [file 12881_2017_485_MOESM1_ESM.docx]

Supplementary table S1. Analysis of SNPs related to CRC.

| **casoc** | Odds Ratio | Std. Err. | z | P>z | [95% Conf. | Interval] |
| --- | --- | --- | --- | --- | --- | --- |
|  |  |  |  |  |  |  |
| rs10795668 |  |  |  |  |  |  |
| 1 | 0.92 | 0.07 | -1.17 | 0.24 | 0.79 | 1.06 |
| 2 | 0.96 | 0.12 | -0.35 | 0.72 | 0.74 | 1.23 |
| rs6687758 |  |  |  |  |  |  |
| 1 | 1.06 | 0.08 | 0.79 | 0.43 | 0.91 | 1.23 |
| 2 | 1.11 | 0.20 | 0.58 | 0.56 | 0.78 | 1.58 |
| rs10936599 |  |  |  |  |  |  |
| 1 | 0.87 | 0.07 | -1.91 | 0.06 | 0.75 | 1.00 |
| 2 | 0.90 | 0.16 | -0.58 | 0.56 | 0.64 | 1.27 |
| rs10505477 |  |  |  |  |  |  |
| 1 | 1.02 | 0.08 | 0.19 | 0.85 | 0.87 | 1.19 |
| 2 | 0.85 | 0.08 | -1.66 | 0.10 | 0.70 | 1.03 |
| rs3802842 |  |  |  |  |  |  |
| 1 | 1.14 | 0.08 | 1.79 | 0.07 | 0.99 | 1.32 |
| 2 | 1.12 | 0.15 | 0.89 | 0.38 | 0.87 | 1.45 |
| rs11169552 |  |  |  |  |  |  |
| 1 | 0.93 | 0.07 | -0.90 | 0.37 | 0.81 | 1.08 |
| 2 | 1.18 | 0.19 | 0.98 | 0.33 | 0.85 | 1.63 |
| rs4444235 |  |  |  |  |  |  |
| 1 | 1.06 | 0.08 | 0.77 | 0.44 | 0.91 | 1.24 |
| 2 | 0.97 | 0.10 | -0.26 | 0.79 | 0.80 | 1.19 |
| rs4939827 |  |  |  |  |  |  |
| 1 | 0.86 | 0.07 | -1.99 | 0.05 | 0.74 | 1.00 |
| 2 | 0.69 | 0.07 | -3.68 | 0.00 | 0.56 | 0.84 |
| rs10411210 |  |  |  |  |  |  |
| 1 | 0.95 | 0.08 | -0.63 | 0.53 | 0.81 | 1.12 |
| 2 | 1.21 | 0.33 | 0.70 | 0.48 | 0.71 | 2.06 |
| rs5275 |  |  |  |  |  |  |
| 1 | 1.01 | 0.07 | 0.08 | 0.94 | 0.87 | 1.16 |
| 2 | 1.05 | 0.13 | 0.41 | 0.68 | 0.83 | 1.33 |
| rs1801282 |  |  |  |  |  |  |
| 1 | 0.92 | 0.09 | -0.87 | 0.39 | 0.77 | 1.11 |
| 2 | 0.92 | 0.42 | -0.19 | 0.85 | 0.37 | 2.25 |
| rs10795668 |  |  |  |  |  |  |
| 1 | 0.92 | 0.07 | -1.17 | 0.24 | 0.79 | 1.06 |
| 2 | 0.96 | 0.12 | -0.35 | 0.72 | 0.74 | 1.23 |
| rs1057910 |  |  |  |  |  |  |
| 1 | 1.16 | 0.12 | 1.43 | 0.15 | 0.95 | 1.41 |
| 2 | 1.06 | 0.48 | 0.13 | 0.90 | 0.43 | 2.58 |
| rs4775053 |  |  |  |  |  |  |
| 1 | 0.95 | 0.08 | -0.55 | 0.58 | 0.81 | 1.13 |
| 2 | 0.90 | 0.26 | -0.37 | 0.71 | 0.51 | 1.59 |
| rs6083 |  |  |  |  |  |  |
| 1 | 1.01 | 0.07 | 0.13 | 0.89 | 0.87 | 1.17 |
| 2 | 1.01 | 0.12 | 0.05 | 0.96 | 0.79 | 1.28 |
| rs762551 |  |  |  |  |  |  |
| 1 | 0.95 | 0.07 | -0.68 | 0.50 | 0.82 | 1.10 |
| 2 | 1.08 | 0.12 | 0.66 | 0.51 | 0.86 | 1.34 |
| rs2470890 |  |  |  |  |  |  |
| 1 | 0.94 | 0.07 | -0.76 | 0.45 | 0.81 | 1.10 |
| 2 | 1.18 | 0.12 | 1.65 | 0.10 | 0.97 | 1.44 |

The minimally adjusted odds ratio (OR) was calculated adjusting by sex, age, educational level. Area, places where cases and controls were recluted, was used as random variable.

Association is evaluated for carrying 1 or 2 SNP minor frequency alleles relative to a reference of zero SNP minor frequency allele.
