# Supplementary material for: The RS4939827 polymorphism in the SMAD7 GENE and its association with Mediterranean diet in colorectal carcinogenesis
Source: BMC Med Genet. 2017 Oct 30;18:122. doi: 10.1186/s12881-017-0485-5 (PMC5661920; doi:10.1186/s12881-017-0485-5)
Supplement: Supplementary file 2 — Associations between polymorphisms and the Mediterranean Diet Pattern of Sofi considering different genetic models. Genetic models: codominant, dominant, recessive, overdominant, log-additive. (DOCX 16 kb) [file 12881_2017_485_MOESM2_ESM.docx]

Supplementary table S2. Associations between polymorphisms and the Mediterranean Diet Pattern of Sofi taking into account different genetic models.

| **Model** | **Genotypes** | **n** | **p-value** |
| --- | --- | --- | --- |
| **Codominant** | T/T | 1116 | 0.17 |
|  | C/T | 1709 |  |
|  | C/C | 669 |  |
|  |  |  |  |
| **Dominant** | T/T | 1116 | 0.41 |
|  | C/T-C/C | 2378 |  |
|  |  |  |  |
| **Recessive** | T/T - C/T | 2825 | 0.18 |
|  | C/C | 669 |  |
|  |  |  |  |
| **Overdominant** | T/T - C/T | 1785 | 0.07 |
|  | C/T | 1709 |  |
|  |  |  |  |
| **Log-additive** | --- | --- | 0.84 |
